# Guaiac Pills for Syphilis

**Published:** 1874-01

**Authors:** A. McBride


Guaiac Pills for Syphilis.—*A. McBride.*—The *success of the pill depends upon how it is made.* If you make the pill with the ordinary excipients, you will fail; for it will be nauseous, and the patient will tire of it. Alcohol, and nothing else, is the only proper excipient. Excuse me if I give you a little lecture on pharmacy. The way to make the pill is as follows: Pulverize the guaiac and sift out ligneous and cortical impurities; then let the operator be in a warm room, have the mortar warm and the pill-machine warm; put the powdered gum into the mortar, add very sparingly of alcohol, beat away, and add more if necessary, but be careful and not get in too much. The object aimed at is to form a mass as stiff as can be worked by means of warmth and a very little alcohol. When the mass is formed, work it rapidly into pills and roll them into a cold tin pan in a cool room. If you make these pills any other way it will prove more or less a failure. By the way, use no pulverised licorice or other powder. If you use never so little too much alcohol, your pill will be soft and never harden.

"Now, of this pill the patient can take from nine to eighteen per day, usually twelve, and will declare he feels better all the time; so much so that if he runs out of pills he will soon call for more. This treatment applies to secondary and tertiary; is excellently adapted to external or cutaneous manifestations.—*Cincinnali Lancet and Observer.*

